# Multidisciplinary team meetings in Hematology: a national mixed-methods study

**DOI:** 10.1186/s12885-023-11431-y

**Published:** 2023-10-07

**Authors:** Alice Polomeni, Dominique Bordessoule, Sandra Malak

**Affiliations:** 1 Clinical Hematology and Cellular Therapy Department, Hôpital Saint-Antoine – Assistance Publique- Hôpitaux de Paris, 184 Rue du Fbg Saint Antoine, 75012 Paris, France; 2 Ethics Commission of the French Society of Hematology, Grenoble, France; 3 Hematology Department, Centre Hospitalo-Universitaire de Limoges, 2 Avenue Martin Luther King, 87000 Limoges, France; 4 Hematology Department of Institut Curie Hospital, Institut Curie-Saint-Cloud, 35 Rue Dailly, 92210 Saint-Cloud, France

**Keywords:** Multidisciplinary team meetings, Care organization, Decision-making, Hematology, Cancer care team, Cancer policy

## Abstract

Multidisciplinary team meetings are a current international practice in cancer care, but to date, few data exist on the specificity of its practice in hematology.

In this manuscript, we present the result of the first national study, realized with quantitative and qualitative methods in France, which brings new insights in order to improve the collegial decision-making process.

To improve the effectiveness of MDTMs, the needs to focus on complex cases, to enhance patient centeredness and teamwork are relevant aspects, and a specific focus on hematological particularities is warranted to truly improve process.

**Background** Understanding the Multidisciplinary team meetings (MDTMs) process in different medical specialties facilitates the identification of core factors supporting effective MDTM work. Our mixed-methods study explores the participants’ perceptions of hematology MDTMs.

**Design** Online questionnaires collected data concerning the decision-making process, benefits and inconveniences of MDTMs for both patients and professionals. Semi-directive phone interviews were conducted and analyzed, thereby supplying qualitative data.

**Results** A total of 205 professionals responded to the questionnaire and 22 participated in the qualitative interviews. The data indicate the unique characteristics of hematology, including a specific definition of collegiality, the frequent solicitation of expert advice and the anticipation of treatment even prior to the occurrence of MDTMs. Additional information concerning patients’ wishes and psychosocial conditions are also needed. Participants emphasize the subjective aspects and the impact of the climate of MDTMs on medical decisions.

**Conclusion** Although MDTMs are recognized to be a valuable tool, organizational and relational issues may interfere with their efficiency.

To improve the effectiveness of MDTMs, the needs to focus on complex cases, to enhance patient centeredness and teamwork are relevant aspects. A specific focus on hematological particularities might be warranted to truly improve the collegial decision-making process in the context of hematology.

**Supplementary Information:**

The online version contains supplementary material available at 10.1186/s12885-023-11431-y.

## Introduction

Multidisciplinary team meetings (MDTMs) are a current international practice to facilitate the care of cancer patients. They bring together health professionals from different disciplines whose skills are essential to establish the diagnosis and to make a decision offering patients the best management according to the state of the medical science. They promote the coordination of communication and treatment, thereby improving the decision-making process and the quality of care [1–5].

In France, the first Cancer Plan (2003–2007) [6] defines MDTMs’ requirements (membership, standards, quorum, procedures and formal reporting criteria for records) and objectives: to standardize the management of cancer patients, analyze medical files and evaluate treatment risks and benefits with the aim of proposing an appropriate decision in accordance with evidence-based medicine (EBM).

According to professionals involved in cancer care in France, MDTMs can improve physicians’ adherence to reference guidelines, inclusions to clinical trials, elaboration of personalized plans of care and the transparency of therapeutic choices. In addition, these meetings play a pivotal role in medical training and the process of updating the relevant knowledge [7–9].

MDTMs are particularly useful in complex cases (e.g., allogeneic hematopoietic cells transplantation – allo-HCT, transition from curative to palliative care decisions), the frequency of which has been estimated to range between 25 and 40% of examined cases [10, 11]. Nevertheless, the time allocated to the discussion of these complex situations during MDTMs may be insufficient due to the significant number of files that must be treated [12–14]. Other impediments of MDTMS, such as poor attendance, insufficient data, lack of administrative support and inadequate leadership, can negatively impact decision-making and the potential effect of MDTMs on the organization of treatment [15].

Research has reported contradictory results concerning the impact of MDTMs on the quality of decision-making and patient outcomes [16–19] and the reported data also show variability in terms of the implementation of the recommendations made during these meetings [3, 20]. Understanding the MDTM process in the context of different medical specialties may contribute to the task of identifying core factors related to effective MDTM work.

To date, little evidence has been collected concerning MDTMs in hematology field [21–24]. In the most extensive data set, including all types of MDTMs, hematology was an outlier in relation to several statements regarding the process of clinical decision-making in MDTMs and its impact on the timeliness of care, as well as on patient choices, involvement, staging and survival rates [25].

Hematological malignancies account for approximately 10% of new cancer diagnoses in Western countries and are the sixth most prevalent form of cancer overall. Intensive and innovative treatments, often potentially lifesaving, are core practices in hematology; however, their toxicities often lead to potentially life-threatening or distressing side effects. Thus, the implementation of MDTMs in the context of hematology may require adjustments to ensure that the modalities of such meetings are able to produce benefits for patients and health care professionals.

Our nationwide, mixed-methods study explores the perceptions of participants in French hematology MDTMs concerning these meetings.

## Methods

### Study design and data collection

We conceived an exploratory study to investigate the representations of health professionals working in Hematology field about the MDTM. A mixed-method approach was chosen to better identify specificities of this specialty: a questionnaire was conceived in the basis of previous research and a qualitative approach was employed to explore relevant points of the survey’s answers.

Our mixed-method study has been performed in accordance with the relevant guidelines [26, 27].

Informed consent was obtained from participants after information about the study’s objectives and methods.

### Quantitative approach and survey

Based on previous research, we developed a 46-item Survey Monkey questionnaire. This online survey software facilitates the collection and analysis of a targeted population’s perception about a subject. Three categories of questions – close-ended, open-ended, and descriptive—may be proposed and the questionnaire may be sent by e-mail and/or web links.

Our questionnaire pertained to the following themes: socio-demographic data, professional profile and a description of MDTMs in terms of respondents’ participation (frequency and kind of MDTM attended), duration, relevant information, quorum, participant professionals, clinical trials inclusions, implementation of MDTMs’ decisions, discussion dynamics, decisional process, patient representation, benefits and inconveniences of MDTMs for patients and professionals. It was composed of different kinds of questions: yes/no questions (e.g. « do you regularly participate in MDTM therapeutic decisions ?»), multiple choice questions (e.g. frequency of MDTMs: weekly/every two weeks/monthly/other); Likert scale questions (e.g. fully agree↔strongly disagree); ranking questions (e.g. from 1 to 5 the importance of data).

Our survey was tested before it was distributed to members of the French Society of Hematology (about 1000 members) and the French Association of Residents in Hematology (about 250 members).

### Qualitative approach and interviews

A qualitative approach was employed to explore certain significant points in the survey, notably respondents’ opinions concerning their participation, the discussion dynamics and the benefits and inconveniences of MDTMs.

The qualitative part of the study has been realized according to COREQ recommendations [27].

We referred to the framework approach [28], according to which relevant themes referred to existing knowledge concerning the topic area, are organized into a pre-established grid. The interview guide was based on the data provided by the Survey Monkey questionnaire. Consenting respondents were contacted to conduct a telephone interview lasting thirty minutes.

A professional with a dual master’s degree in psychology and sociology who also had twenty years of experience in clinical and research work in the field of hematology conducted all the interviews, which were recorded and transcribed in their entirety.

### Data analysis

Quantitative data were processed only by descriptive statistics, reported by raw numbers and percentages for simple and multiple-choice questions, weighted average was added to Likert scale and ranking questions. No comparisons were made among different subgroups. As all respondents did not answer to all the questions, we reported the number of respondents for each question, so to avoid wrong interpretations of data.

NVIVO 8 computer-assisted qualitative data analysis software was used to assist in the handling, storage and management of the data. Two investigators coded the transcripts independently: each investigator used tree nodes to organize codes into a hierarchical structure and subsequently established relationships among different themes and subthemes. A third investigator reviewed the coding process and participated in the thematic analysis. The interpretations provided by the three investigators were than validated by other collaborators (consensual validity).

## Results

### Quantitative data

Of the 205 respondents, ranging from 26 to 68 years old, 58% were women. They mostly work in France (93%): 67% in clinical hematology services, and 74% in university hospital centers. Most of respondents were clinical hematologists (67%) and biological hematologists (22%). See Table 1 for more details regarding the survey’s population.

**Table 1:** Socio-demographic and professional profile of survey’s respondents

Variables	
Number of respondents n. (%)	205 (100%)
Gender n. (%)	
Female	117 (58%)
Male	84 (42%)
Missing/unknown	4 (2%)
Age median (range)	40 (26–68)
Specialties n. (%)	
Clinical hematology	135 (67.5%)
Biological Hematology	45 (22.5%)
Mix activity: clinical and biological hematology	7 (3.5%)
Medical oncology and hematology	8 (4%)
Internal medicine	5 (2.5%)
Missing/unknown	5 (2.5%)
Place of practice, n. (%)	
University hospital	146 (74%)
Regional hospital	6 (3%)
Local hospital	7 (12%)
Comprehensive cancer center	8 (7%)
Private hospital	5 (2,5%)
Missing/unknown	6 (3%)
Country of exercise, n. (%)	
France	186 (93,5%)
Algeria	3 (1,5%)
Moroco	5 (2,5%)
Togo	2 (1%)
Tunisia	1 (0,5%)
USA	1 (0,5%)
Haiti	1 (0,5%)
Missing/unknown	6 (3%)

One hundred fifty-four complete responses were ultimately obtained, but all answers are reported: number of responses and percentage are indicated for each item. For significant quantitative data please see Additional file 1.

Among the respondents, 93% participated in one or more MDTMs, mostly local (80%) or inter-hospital meetings (43%) in generalist hematology (64%), lymphoid pathologies (57%), myeloid pathologies (54%) and transplantation (39%). The mean length of the MDTMs was 120 min, and about 20 files were reviewed in average during each meeting.

Opinions were divided concerning the specialties that should represent a minimum quorum for a hematology MDTM: for 32% of respondents, 3 physicians with different specialties (clinician, biologist or radiologist) including at least a clinical hematologist were necessary; whereas for 24% of respondents, 3 clinical hematologists were required. The participants’ specialties most frequently cited were clinical hematologists, biological hematologists, cytogeneticists/molecularists.

Regarding patient centeredness, 92% of respondents (61% always and 31% frequently) informed the patient that his/her file was to be discussed among colleagues, but only 14.6% and 13.4% considered patient preferences and psychosocial data to be essential elements involved in producing an optimal therapeutic proposal, respectively. It is important to note that if the treatment implemented is different from that proposed by the MDTM, only 54% of the respondents systematically notified patients about this decision.

Most of respondents believed that MDTMs contribute to enhance decisions in line with the relevant guidelines (95%), to increase inclusion in clinical trials (91%), to improve decision-making (100%), care coordination (89%), quality of care (92%) and feeling of safety for patients (92%). Regarding the MDTMs’ role in improving patient prognosis, timeliness of exams or treatments and patient involvement in medical decision-making, respondents’ opinions were more divided.

About the benefits of MDTMs for professionals, respondents are unanimous about the advantages related to information/knowledge sharing (100%), interactions with colleagues (100%), reviewing the patient file (99%) and helping with decision-making (98%). Other benefits, such as reducing decision uncertainty, sharing legal responsibility and increasing work satisfaction are mentioned.

**Fig. 1:**
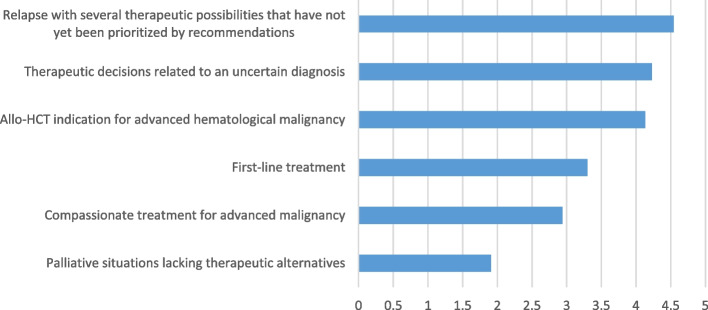
Situations where MDTMs discussions appears particularly useful

The clinical situations most frequently discussed in hematology MDTMs are: hematological malignancies (95%), difficult diagnoses (83%), benign complex cases (54%), and malignant cases without therapeutic indication (e.g., stage A CLL) (41%). MDTMs discussions appears to be particularly useful with respect to the situations outlined in Fig. 1, ranked from most to least useful: relapse with several therapeutic possibilities that have not yet been prioritized by recommendations; therapeutic decisions related to an uncertain diagnosis; allogeneic hematopoietic cell transplantation (allo-HCT) indication for advanced hematological malignancy; first-line treatment; compassionate treatment for advanced malignancy; and palliative situations lacking therapeutic alternatives.

Respondents were asked to rank suggestions in order of usefulness of discussions; averages were assigned to each answer choice (1 = least useful, 6 = more useful).

Weighted average of clinical situations discussed in hematology MDTM provided by respondents (*N* = 105).

For more than 98% of respondents, the treatment can be initiated prior to MDTM discussion. This situation can happen in up to 21% of the cases, corresponding to therapeutic emergencies.

Eighty-seven percent of the respondents often sought advice from external experts, as frequently as 10 times per month. The reasons why MDTMs may not be adequate to produce a therapeutic proposal are (ranked from most frequently cited to least frequently cited): the need to consult references or seek expert advice (71%), a lack of information regarding the pathology (70%) or the patient (57%), the complexity of the case (48%), a disagreement among MDTM participants (14%).

The disadvantages of MDTMs are reported less often but the following are quoted: technical issues (55%), interrupts/delays (51%), exacerbation of problems within teams (36%), time losses (27%), peer judgments (15%), and the fact that such meetings are likely to lead to insufficient consideration of patient preferences (25%). Only 6% of respondents considered MDTM to be inappropriate in the context of hematology.

### Qualitative data

Twenty-two of the survey respondents agreed to participate in the qualitative study. They were mostly hematologists (99%) and men (59%) between the ages of 28 and 65. They worked mainly in adult clinical hematology departments (77%) or oncology-hematology units (23%) as public hospital practitioners. They participate weekly at least to a local MDTM; they may also take part in specific pathology and/or regional MDTM.

For more details about qualitative study’s respondents see Table 2.

**Table 2:** Qualitative study’s respondents: socio-demographic, professional profile and attendance to MDTM

**ID**	**Age** (y)	**Sex** (F/M)	**Specialty**	**Place of practice**	**Type of MDTM/Frequency**
1	33	M	Hematologist	Clinical Hematology Local Hospital	Regional/weekly
2	47	M	Hematologist	Oncology-Hematology Private Hospital	Local/weekly Inter-hospitals/3 times a week
3	29	M	Hematology Resident	Clinical Hematology University Hospital	Local/weekly Inter-hospitals /bimonthly
4	32	F	Pharmacist	Oncology-Hematology University Hospital	Local/weekly
5	28	F	Hematology Resident	Clinical Hematology University Hospital	Local/bimonthly Inter-hospitals/bimonthly
6	57	M	Hematologist	Clinical Hematology Regional Hospital	Inter-hospitals /weekly
7	58	F	Hematologist	Oncology-Hematology University Hospital	Local/weekly
8	36	F	Hematologist	Hematology Local Hospital	Local/weekly
9	61	F	Hematologist	Clinical Hematology Local Hospital	Local/weekly Inter-hospitals /bimonthly
10	32	F	Hematologist	Clinical Hematology Local Hospital	Local: 3 times a week Inter-hospitals/monthly
11	44	M	Hematologist	Clinical Hematology Regional Hospital	Local:/3 times a week Inter-hospitals: monthly
12	46	M	Hematologist	Clinical Hematology University Hospital	Local: weekly Inter-hospitals/3 times a week
13	48	F	Hematologist	Oncology-Hematology University Hospital	Local: weekly
14	46	M	Hematologist	Clinical Hematology Comprehensive cancer center	Local: weekly Inter-hospitals: monthly
15	63	F	Hematologist	Clinical Hematology University Hospital	Local/ 3 times a week Inter-hospitals/ monthly
16	45	M	Hematologist	Clinical Hematology University Hospital	Local/ 3 times a week Inter-regional/monthly
17	43	M	Hematologist	Clinical Hematology University Hospital	Local/weekly
18	64	M	Hematologist	Clinical Hematology University Hospital	Local/weekly Inter-hospitals/bimonthly
19	65	M	Hematologist	Clinical Hematology University Hospital	Local/weekly Inter-hospitals/weekly
20	33	F	Hematologist	Clinical Hematology Regional Hospital	Local/weekly
21	33	M	Hematologist	Oncology-Hematology University Hospital	Local/weekly Inter-hospitals/weekly
22	30	M	Hematology Resident	Clinical Hematology University Hospital	Local/weekly

Qualitative data were organized in accordance with the following themes: MDTM implementation, the dynamics of the MDTM (organizational aspects and subjective issues), the discussion of complex cases, the failure to implement the MDTMs’ decisions, the benefits and inconveniences of MDTMs and suggestions for improving MDTMs.

### MDTM implementation

Most respondents mentioned the preliminary existence of weekly “staff” meetings prior to the implementation of MDTMs as encouraged by the French Cancer Plan. In their opinion, the current regulatory status of the MDTMs, which required the presence of various specialists to ensure the collective character of decisions made during these meetings, served as a guarantee against unilateral decisions.

### The dynamics of the MDTM

Most interviewees highlighted the issue of MDTM dynamics, which depend on both organizational and “subjective” aspects (see Table 3 for significant quotations).

**Table 3:** MDTM dynamics. Significant quotations from the qualitative interviews. The respondent’s identifications (referring to Table 2) are in brackets

Decision-making versus training task
“When there are too many participants, (…) I am not sure that it is useful, we waste time, because people who don’t know anything about the pathology are going to intervene… It is true that it may have an interest in medical training, but the main objective is to reach a medical decision” [ID 16]
**Quality of discussions**
“I know that the recording is compulsory, but I think that MDTM loses some of its meaning by taking time for registration because we need more time for complex situations” [ID 22] “When the patient is beyond the therapeutic resources, the discussions can be stormy” [ID 22] “At first, we need a good knowledge of the files, but generally, the files are presented very quickly and this is problematic! (…) I think it would be better to postpone the non-urgent files to the week after, rather than trying to present everything” [ID 9]
**Team’s dynamics**
“It is really a kind of mini concentration of the team’s life, with people who speak systematically, while others never speak and put themselves in the background, and some who even may show some hostility” [ID 8] “We realize that the MDTM is a place of exchange, I do not think that it is to score-settling meeting in our Department. But it doesn’t mean that there is no conflict…” [ID 13]
**Taking part on the decisions**
“Globally, we feel comfortable to speak as long as we have an area of competence” [ID 11] “There are two categories of people in MDTM: there are the people who speak on one side and who express an opinion which holds place of standard, in the name of an expertise, and the others, who listen…” [ID 19]
**« Atmosphere»**
“Each one is free to express an opinion, (…) even the residents, biologist takes their place, everybody manages to discuss the files. MDTM are a moment of exchanges that we appreciate all because it is also a training setting” [ID 7] “There were hostilities within the team, which led to stuck discussions, to peremptory decisions, the debates were immediately closed, there was no possible exchanges”. [ID 3] “I find that MDTM tends to function in a partial way: there are one or two people who impose their decisions on others. I think that the listening is not always open…the decision-making process is not really collegial…” [ID 5]
**Participants’ involvement**
“Personally, I try to be active in these meetings, participative, and my colleagues also: each participant can express his/her opinion about the decision to be taken” [ID 18] “A physician presents his/her file, some colleagues don’t pay any attention and continue to mind their own business: we make the MDTM in front of our computers, and sometimes you have the impression that some colleagues are looking at their e-mail…” [ID 8]
**The role of the moderator**
“In this Department, it is the Department head who decides, so, when he is not there, we don’t make choices because we know that he will send an email the next day changing the MDTM’s decision” [ID 22] “I attended MDTM in different Departments where the Department head was being is a little bit dictatorial, someone who imposed his point of view: this attitude blocked any word, there was a sort of judgment on the person presenting the file and others don’t dare to speak… it is very difficult” [ID 19] “In our Department, it is the Department Head who is the moderator; he has the intelligence to encourage everyone to participate, and he has the modesty to say that if there is another expert of this disease in the room, "it is not me who is necessarily right” [ID 1]
**The « final word»**
“There is a technical aspect (the bibliography, the protocols) and the experience aspect and it is true that for me, the Department Head, because of his experience and his knowledge of the specialty, should have the final word.” [ID 6] “It is the referent hematologist (RH) who has the final word. I am the Department head and I often lead the MDTM but even when I don’t agree with the RH’s point of view, I maintain that it is the RH who has the final word” [ID 16] “I think that between what is decided in MDTM and what is really implemented, there may be gaps… and if it is the RH who decides, it is not necessarily a standardized choice, thus, there is a subjective bias…” [ID 20]

### Organizational aspects

The number of MDTMs’ participants ranged from 5 to 20. The interviewees highlighted their interest in the presence of various specialists, but they also wondered whether too many participants could influence the decision-making process negatively.

In addition to the medical data, the presence of the referent hematologist is essential: he or she is supposed to be aware of patients’ conditions, preferences and psychosocial data, which are rarely recorded in medical files. This information is particularly useful when the efficacy of different therapeutic options is equivalent. It is worth noting that some interviewees questioned the influence of these “subjective data”, which may be affected by the referent hematologist’s perceptions, on decisions made during MDTMs.

The time allocated to the task of examining files influences the quality of the discussions, which also depends on whether the cases are examined at the beginning or the end of the meeting.

Most interviewees considered the “compulsory recording” of all files to be “a waste of time” and believed that "difficult cases" should be prioritized. Other interviewees highlighted the risk of neglecting “simple files”, which could nevertheless raise important decisions, such therapeutic abstention. Some respondents also emphasized the importance of reminding all MDTM participants of current recommendations. Regarding this issue, other interviewees mentioned time pressure and drew attention to the discrepancy between the purpose of MDTMs, i.e., to make decisions, and the pedagogical value of such meetings.

### Subjective issues

Several interviewees described an MDTM as a “mini-concentration of the team’s life”. Participating in MDTM discussions is correlated to the participant’s expertise and to the ambience of the meeting. A “kind ambience” facilitates the expression of opinions by all participants. In contrast, "interpersonal conflicts" may emerge during these meetings, thereby hindering these discussions and leading to "peremptory" positions. Some interviewees noted that two groups participate in an MDTM: “professionals who express an opinion that exudes authority and those who listen…”.

The ambience of an MDTM is associated with the team’s interpersonal relationships and the role of the department head, who is typically the moderator of these meetings. Two "figures" are described: a moderator who facilitates speaking and stimulates exchanges by adopting a position of equality with regard to each participant and a leader who imposes his or her recommendations, returning to decisions made in his or her absence and discouraging the participation of the team members.

### Discussing complex files

The interviewees mentioned “complex files”, which could lead to a demanding decision-making process or even occasionally to the absence of a unanimous therapeutic proposal. Aspects of the situations discussed included the following:

- Doubts regarding diagnosis
- Therapeutic abstentions
- Multi-treated patients
- Comorbidities jeopardizing the feasibility of standard treatments
- Inclusion in new clinical trials
- Prescription of new, expensive treatments
- Indications supporting Hematopoietic Cell Transplantation (HCT)
- Palliative care (PC) decisions.

Indications suggesting HCT and PC decisions were quoted most frequently as “complex situations”, which involved a significant risk of "unreasonable stubbornness". Participants noted that information regarding patients’ psychosocial situations and wishes was rarely presented.

Several interviewees questioned the overestimation of the benefits of curative treatments in the context of these medical situations and observed that the advice given during MDTMs frequently lacked the power to alter the initial convictions of the referent hematologist.

When a consensus was not reached, two or three therapeutic alternatives could be proposed to the referent hematologist. Some interviewees noted that "subjective" bias could interfere with the referent hematologist’s decision. These interviewees evoked the possibility of resorting to asking experts on specific pathologies, reference centers focusing on rare diseases or even national-/expert-level MDTMs. Other interviewees affirmed that the “final word” should be given to the department head due to his or her wealth of scientific knowledge and clinical experience. Some respondents also remarked that, in all cases, “the decision should be shared with the patient”.

### The failure to implement MDTMs’ decisions

The reasons for the failure to implement decisions made at the MDTM were as follows: a lack of medical elements, the absence of the referent hematologist and/or the pathology specialist, the patient’s refusal and the evolution of the patient’s clinical situation. In these latter situations, either the file was presented at the next meeting (occasionally a regional and more expert MDTM) or the referent hematologist made a decision based on EBM recommendations.

A “really exceptional situation” occurred when the referent hematologist disagreed with the conclusion of the MDTM, instead opting for another therapeutic choice. These situations were associated with tense interpersonal relationships and MDTM dynamics involving conflict.

### Benefits of MDTMs

Most interviewees were rather enthusiastic regarding the benefits of the MDTMs and mentioned the following aspects: the benefits for training and updating one’s knowledge, homogenization of practices, legitimization of decisions and shared responsibility (see Table 4 for significant quotations in this context).

**Table 4:** MDTM benefits. Significant quotations from the qualitative interviews. The respondent’s identifications (referring to Table 2) are in brackets

Training and Updating on the knowledge
“I think that MDTM has an educational function, and not only for the medical residents, for the seniors also”. [ID14] “In a General Hematology Department, we do not have the possibility to get informed about all the publications relatives the different hematological malignancies and as each physician develops an expertise, each one of us can learn something during MDTM”. [ID 12]
**Legitimization of the decision**
“I think that the collective decision is rather positive: for the doctor who is alone and who needs an opinion, but also for the patient who knows that there are several physicians who thought about his/her case”. [ID 4]
**Homogenization of medical practices**
“MDTM avoid having heaps of treatments which depend on each physician’s opinions. For example, if a physician has the tendency to frequently prescribe palliative care, MDTM may propose still feasible curative treatment. Likewise, when a physician tends to propose systematically a curative treatment even after several treatments in pejorative prognosis’ pathology, the discussion may counter this tendency” [ID 10]
**Physicians’ security**
“Working in a standardized setting which guarantees the collective back up in case of contentious situations with the patient and/or his/her family is a real benefit for the physician”. [ID 2]
**Sharing responsibility**
“I think that our practice is extremely difficult because we must take decisions that impact the patient’s life. MDTM reassure me: validating my decisions with my colleagues, asking their expertise helps me in my daily practice”. [ID 8] “I remember when the decisions were taken by the physician alone, and I wouldn’t like to go back to this situation. When everything works, then you can be very proud of yourself; conversely, when problems arrive, you are alone and worried”. [ID 21] “Initially, I thought that the MDTM would come to tarnish the image that the patient has of the doctor (“the holder of a knowledge, susceptible to save lives”); currently, the MDTM helps me facing therapeutic uncertainties”. [ID 13]
**Benefits for patients**
“In our Department, there is a significant recruitment to clinical trials. MDTM allow us to be informed about new protocols and that is very interesting for our patients”. [ID 4] “For the patient, the homogenization of medical practice is a real advantage, so he/she does not depend on the opinion of a single doctor. It is about equity of chances!” [ID 5] “It is reassuring for the patients to know that the decision is collegial and validated by all the Department’s medical team”. [ID 1]

MDTMs are a mean of regulating individual practices and, in the event of litigations or legal actions, may be used as a “safeguard” for physicians. Other benefits of MDTMs include the positive consequences for the organization of clinical activities and team cohesion.

In the opinions of respondents, the most important benefit to patients was the discussion of their files by a panel of experts, thus increasing the medical relevance of the resulting therapeutic choice. In addition, MDTMs can increase patients’ chances of benefiting from the latest therapies, of being included in clinical trials and of accessing scientific advances in various specialties. A few respondents also emphasized ethical arguments relating to equality of opportunity due to the homogenization of medical practices.

### Challenges to the effectiveness of MDTMs

Significant quotations concerning this point are provided in Table 3.  The most frequently quoted inconvenience was the fact that MDTM is “very time-consuming” and that institutions do not allocate specific time to engage in this activity.

Several interviewees regret the influence of interpersonal conflicts on the decision-making. Others criticize the "mechanical character” of decisions based only on scientific knowledge, notably neglecting the psychosocial aspects of clinical situations.

The MDTM’s interference in the doctor–patient relationship is particularly noticeable when the collective decision conflicts with the perspective of the referent hematologist. Some interviewees informed patients that their initial therapeutic suggestion would be examined in MDTMs prior to a definitive decision being made.

The majority of interviewees did not report any inconveniences of MDTMs for patients. Some interviewees noted that therapeutic decisions concerning "very advanced" diseases are not always suitable for the patient’s situation due to a lack of psychosocial data (see Table 5 for significant quotations in this context).

**Table 5:** MDTM’s disadvantages. Significant quotations from the qualitative interviews. The respondent’s identifications (referring to Table 2) are in brackets

I. Disadvantages for the teams
**I.1.Time**
“The problem is the allocated time, because the number of files has increased, recording is compulsory and we need more time to discuss complex situations”. [ID 15] “The preparation time is substantial, but it is counterbalanced by the fact that it allows us to make a synthesis of the file”. [ID 14]
**I.2. Participants’ involvement**
“What unpleasant it can be when a colleague answers the phone or is not concentrating: we have important decisions to make!” [ID 7]
**I.3. Quality of discussions**
“The current MDTM carries the risk of being more « mechanical», we tend to apply protocols, and forget the singularity of a given situation (…), as co-morbidities, the patient’s context of life …”.[ID 19] “There is a whole aspect of the management of the patient, which is forgotten (…) I think that discussions are limited, based on partial vision because there is only the medical approach”. [ID 3]
**I.4. Limits**
“In complex situations, notably in palliative decisions, most of the time the referent hematologist’s (RH) point of view will not be modified by MDTM discussions”. [ID 11] “When a consensual proposition is not found, the RH takes the decision. But the problem is that such doctor is going to prefer the treatment X because he had bad experiences with the treatment, whereas another doctor, who have had negative experiences with the treatment Y, will prefer the treatment X… it is too subjective!”. [ID 20]
**II. Disadvantages for patients**
“For the patient, it is not so reassuring to know that his/her doctor chooses a treatment, whereas another doctor would make other choices, it is not very standardized, as a result, it is a random decision…”.[ID 5] “I think that in complex situations, some MDTM decisions are not fit to the patient because psychosocial data are not taken into account”. [ID 6]

### Improving MDTMs

Respondents emphasized the fact that institutional recognition of MDTMs as a medical activity is essential to the improvement of these meetings.

Most interviewees’ suggestions concerned logistics: adequate support (appropriate for the specific situation of hematology) and human resources, particularly with respect to secretaries.

Other suggestions concerned organizational matters, notably time regulation: some respondents proposed a preliminary "sorting" of priority files to limit the cases to be discussed during the meetings so that all these cases could be treated equitably.

In addition to the need for the regular participation of pathologists, cytologists, radiologists, and pharmacists, some respondents also suggested the inclusion of other medical specialists (geriatricians and/or PC physicians) or paramedical professionals (nurses, social workers, or psychologists). Other respondents recommended the inclusion of multidisciplinary staff for discussions regarding difficult files, in particular cases in which PC was indicated.

The role of the moderator in limiting interference (e.g., outside requests or phone calls) and encouraging discussion was emphasized. Two interviewees suggested that MDTM moderators should have training in group-leading techniques.

## Discussion

Our study is the first nationwide, mixed-methods study to explore the perceptions of participants in hematology MDTMs.

Some results seem to be specific to the decision-making process associated with hematology.

The frequent solicitation of external experts reflects the complexity of hematologic practice, given the existence of hundreds of different hematologic malignancies and the need for accurate and specific expertise to ensure the best possible therapeutic proposal.

In fact, our respondents seem to value the benefit of collegiality more in the contexts of complex cases and difficult situations, even with respect to the management of benign diseases or difficult diagnoses, than in the context of the systematic discussion of every new cancer case, which is actually the regulatory requirement in most countries [29].

With respect to other kinds of tumors, core participants in the MDTMs usually include medical oncologists, radiotherapists and surgeons. In hematology, the therapeutic aspect relies exclusively on clinical hematologists, and while others in attendance are considered to provide substantial help regarding accurate diagnosis and staging, they are frequently less involved in the choice of treatment itself. This situation leads to a particular definition of the MDTM composition required for hematology, which, moreover, can vary depending on the more specific type of disease (e.g., generalist, lymphoid, myeloid) or treatment (auto or allo-HCT, car-T-cells therapy).

Moreover, the frequent anticipation of treatment even prior to discussion at an MDTM in the context of rapidly progressive diseases (such as acute leukemia or aggressive lymphoma) seems to be a particular aspect of hematological practice.

Besides hematology specificities, our study highlights relevant points regarding MDTMs’ decisional process and ethical issues, which may also concern other medical specialties.

As noted in the literature, essentially in the contexts of boards pertaining to solid tumors, our results show that the decision-making process associated with MDTMs is affected by organizational and interpersonal factors [4, 11, 15, 19].

The most frequently quoted organizational factor is the insufficient time allocated to the tasks of preparing for the meeting and to examining all files accurately. Variations in terms of the quality of file discussion and insufficient time for in-depth discussion of complex files are relevant issues that have been described previously [3, 8, 13]. Regarding this issue, a French retrospective analysis of MDTM data highlighted the conflict between the need to respond to the completeness required by the Cancer Plan and the importance of prioritizing multidisciplinary [9]. As other studies have shown [2], the absence of institutional acknowledgment is also considered to be an obstacle to MDTM efficiency: organizational factors impact participants’ attendance negatively, which is a primary reason for the failure to implement MDTM decisions. Among the reasons for the failure to implement MDTM recommendations, our results indicate patient refusal, which may be attributed to poor consideration of patients’ choices and their psychological and social conditions throughout the decision-making process [13, 30–34].

Our data highlight the relevant issue of the usefulness of psychosocial information.

Participants seem to be ambivalent regarding this subject: quantitative data show that a quarter of the respondents consider that patient preferences are insufficiently took into consideration, but less than 15% think that neither these preferences nor psychosocial data are essential to make therapeutic choices. Qualitative data contribute to understand this point: psychosocial data seem to be considered relevant in clinical situations that entail a significant risk of "unreasonable stubbornness" (e.g., allo-HCT indications) or in cases in which a PC decision could be discussed.

Due to the paucity of psychosocial data in medical files, the issue of the subjective perception of the patients' conditions and preferences by the referent hematologist has been raised. To address this issue, the participation of nurses and other health care professionals and PC teams in MDTMs is suggested [10], and such participation has already been implemented in some countries [19]. Nevertheless, some evidence shows that the inclusion of nursing personnel is viewed as less important [14, 35–37]. Indeed, several authors have shown that the biomedical approach tends to take precedence over other points of view [15, 35, 38, 39].

The decision-making process is influenced by MDTM dynamics, which are themselves affected by the number of participants involved in the meetings, team relationships and the performance of the MDTM coordinator.

The group’s size is related to its diversity and range of abilities, but it may negatively affect the equality of participation and the effectiveness of the decision-making process [11]. Regarding the number of participants, our data highlight the issue of a possible discrepancy between two main tasks of an MDTM: the decision-making process and the goal of training and knowledge updating.

Team relationships and the coordinator role influence the involvement of the participants, the decisions made at the MDTM and, indirectly, the implementation of those decisions. Indeed, the internal elements of these groups (cultures, beliefs, attitudes, and the interactions among group members), interpersonal factors (lack of trust or respect between team members) and hierarchical stances can impede the decision-making process. It has been shown that team dynamics, ranging from interactive debates to exchanges dominated by single individuals, may impact interactivity and MDTM discussions [14, 37].

Our qualitative data highlight the participants’ concerns about the influence of subjective factors on clinical decisions. A recent French study, based on an ethnographic observation of MDTMs related to breast and ovarian cancer and referred to Longino’s theory of scientific deliberation [40] showed that MDTMs do not always respond to the conditions required to ensure objectivity and rationality. Nevertheless, the author noted that the lateral control among peers and the collective evaluation of the most recently available data contribute to limiting the influence of subjective preferences in the MDTM setting [41].

The meeting climate and quality of the decision-making process both impact the participants’ opinions regarding recommendations made at the MDTM. Some evidence shows that approximately one-third of MDTM participants disagree regarding the decisions ultimately made at such meetings [42]. This unexpressed dissent influences the participants’ feelings concerning the way in which the MDTM can interfere with doctor–patient interactions, although other research has shown that MDTMs do not affect this relationship [43].

These issues regarding MDTM dynamics and the pivotal role of the MDTM coordinator have been acknowledged [43–47]. The role of personal qualities and nontechnical skills in managing MDTMs has been emphasized, and specific leadership training for MDTM chairs has been recommended [3, 48, 49].

In spite of organizational and interpersonal issues, hematologists – as professionals of other medical specialties, seem to highly appreciate MDTMs and largely recognize its value as part of the decision-making process.

### Limitations

Our study faces certain limitations, notably with respect to the sampling process. Regarding quantitative data, online survey monkey don’t allow researchers to know if concerned population (SFH and AIH members) received e-mails asking their participation. Besides, a quarter of the respondents didn’t answer to all the questions, negatively impacting the analysis of the data.

Concerning the qualitative study, we consider that voluntary participation may induce a bias connected to the particular views of the respondents with respect to the subject in question.

Moreover, even if the similarities are noted between the population of the survey and the qualitative study regarding the medium age (40 years old) and the frequency of participation in MDTM (weekly), the interviewed professionals are not representative of the survey’s population. The sex ratio is inverted: 58% of the survey’s participants are female, whereas in the qualitative study, most of the respondents (59%) are male. It is difficult to know if gender differences may impact the professionals’ perception of the approached themes.

As for the survey’s respondents, the majority of qualitative study’s respondents work in university hospitals (respectively 74% and 55%), but the percentages of respondents working in local and regional hospitals were higher in the qualitative study. We may make the assumption of the influence of the work conditions in these different institutional settings on the respondents’ appreciations of MDTM issues, but we didn’t explore this point.

Further research could explore the means to improve the quality of MDTM and the decision-making process in hematology. Taking into account the need for frequent referral, a particular definition of multidisciplinarity and consideration of frequent emergencies might be warranted to truly improve the collegial discussion proceeding in this medical specialty.

Public policies considering the unmet needs of MDTMs could be envisaged, such as better recognition of the time dedicated to this activity and its preparation, technical issues and specific leadership training for MDTM chairs.

## Conclusion

Our study is the first nationwide, mixed-methods study to explore the perceptions of participants in hematology MDTMs. It highlights certain aspects of the decision-making process in this medical specialty, such as the frequent need for referral, the particular definition of the participants’ specialties and the recurrent clinical emergencies. But it also points out organizational and interpersonal issues, which may interfere with MDTMs’ performance.

Organizational obstacles are mainly related to a lack of institutional recognition of this medical activity (in terms of time pressure and workload) and to the compulsory registration of all files. Poor team ambience negatively influences MDTM discussions and the implementation of its recommendations. A main result of our qualitative study underscores the influence of subjective factors on clinical decisions, which are expected to adhere to scientific data and EBM. These findings raise important ethical issues, which should be explored by larger research – not only in the Hematology, but also in the context of other medical specialties.

### Supplementary Information

**Additional file 1.**

## Data Availability

The quantitative data used to support the findings of this study are available at Sandra Malak, Alice Polomeni, 2022, French Multidisciplinary meetings in hematology, Synapse storage, DOI: https://doi.org/10.7303/syn35106206. The qualitative data used to support the findings of this study are restricted by the Ethics Commission of the French Society of Hematology in order to protect participants’ privacy. Data are available from Alice Polomeni, alice.polomeni@aphp.fr for researchers who meet the criteria for access to confidential data.
