# Supplementary material for: Multidisciplinary team meetings in Hematology: a national mixed-methods study
Source: BMC Cancer. 2023 Oct 7;23:950. doi: 10.1186/s12885-023-11431-y (PMC10560417; doi:10.1186/s12885-023-11431-y)
Supplement: Supplementary file 1 — Additional file 1. [file 12885_2023_11431_MOESM1_ESM.docx]

**Table 2 - Quantitative data**

| **MDTMs’ characteristics** | | |
| --- | --- | --- |
| MDTM’s hematology subspecialty, n. (%)  General hematology  Lymphoid pathologies  Myeloid pathologies  Bone marrow transplant  Specific to certain pathologies  Benign pathologies  Multi-professional  Mixed Hematology and oncology  Other | 179 responses  114 (64%)  103 (57,5 %)  96 (54%)  71 (39%)  33 (18%)  26 (14,5%)  14 (8%)  4 (2%)  16 (9%) | |
| MDTM’s type, n. (%)  Local  Inter-hospital  National (teleconference)  Mail  Other | 183 responses  144 (80%)  77 (43%)  27 (15%)  2 (1%)  7 (4%) | |
| Frequency of MDTM, n. (%)  Weekly  Bimonthly  Monthly | 182 responses  180 (88%)  12 (12%)  7 (7%) | |
| Nb of clinician hematologist per MDTM, median (range) | 162 responses  6 (1-36) | |
| Duration of MDTM (min), median (range) | 120 (45-240) | |
| Number of cases presented, median (range) | 20 (5-40) | |
|  | | |
| **Minimal quorum requested in hematology MDTM** | | |
| Proposed Answers: | 166 responses/ n. (%) | |
| 3 physicians with different specialties (clinician, biologist or radiologist) including at least a clinician hematologist | 53 (32%) | |
| 3 clinician hematologists | 40 (24%) | |
| 3 physicians with experience in Hematology including at least a clinician hematologist | 27 (16%) | |
| 2 clinician hematologists and 1 biologist | 18 (10%) | |
| 1 hematologist, 1 biologist, 1 radiologist | 18 (10%) | |
| 1 hematologist, 1 radiotherapist, 1 surgeon | 0 (0%) | |
| Other | 10 (6%) | |
|  | | |
| **Participants actively involved in therapeutic discussion** | | |
| Respondents were asked to rank professionals’ involvement; averages were assigned to each answer choice (1= never, 5 = always) | | |
| 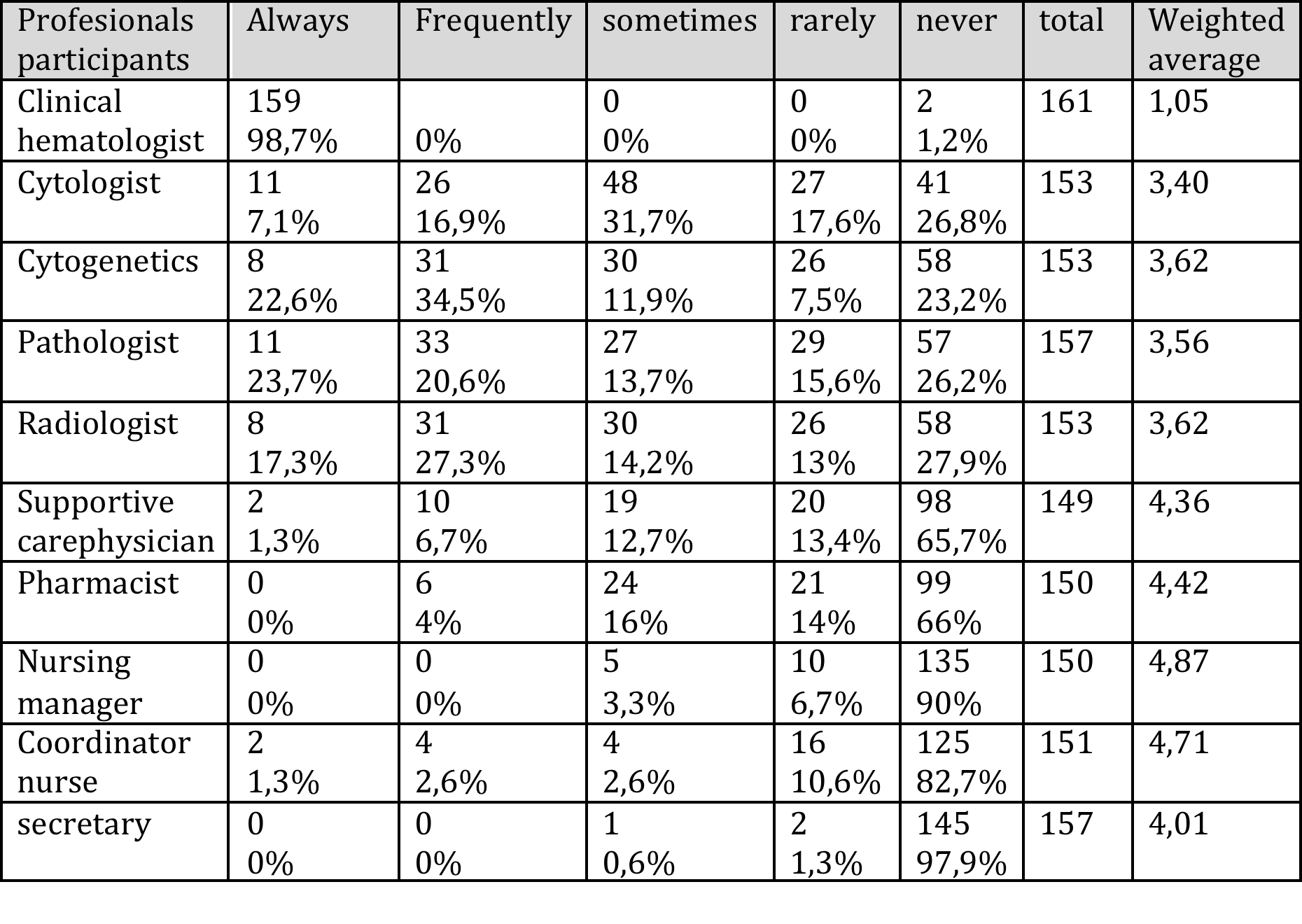 | | |
|  | | |
| **Clinical situations benefiting the most from the discussion in MDTM** | | |
| Respondents were asked to rank proposed answers; averages were assigned to each answer choice (1= least useful, 6 = more useful) | | |
| 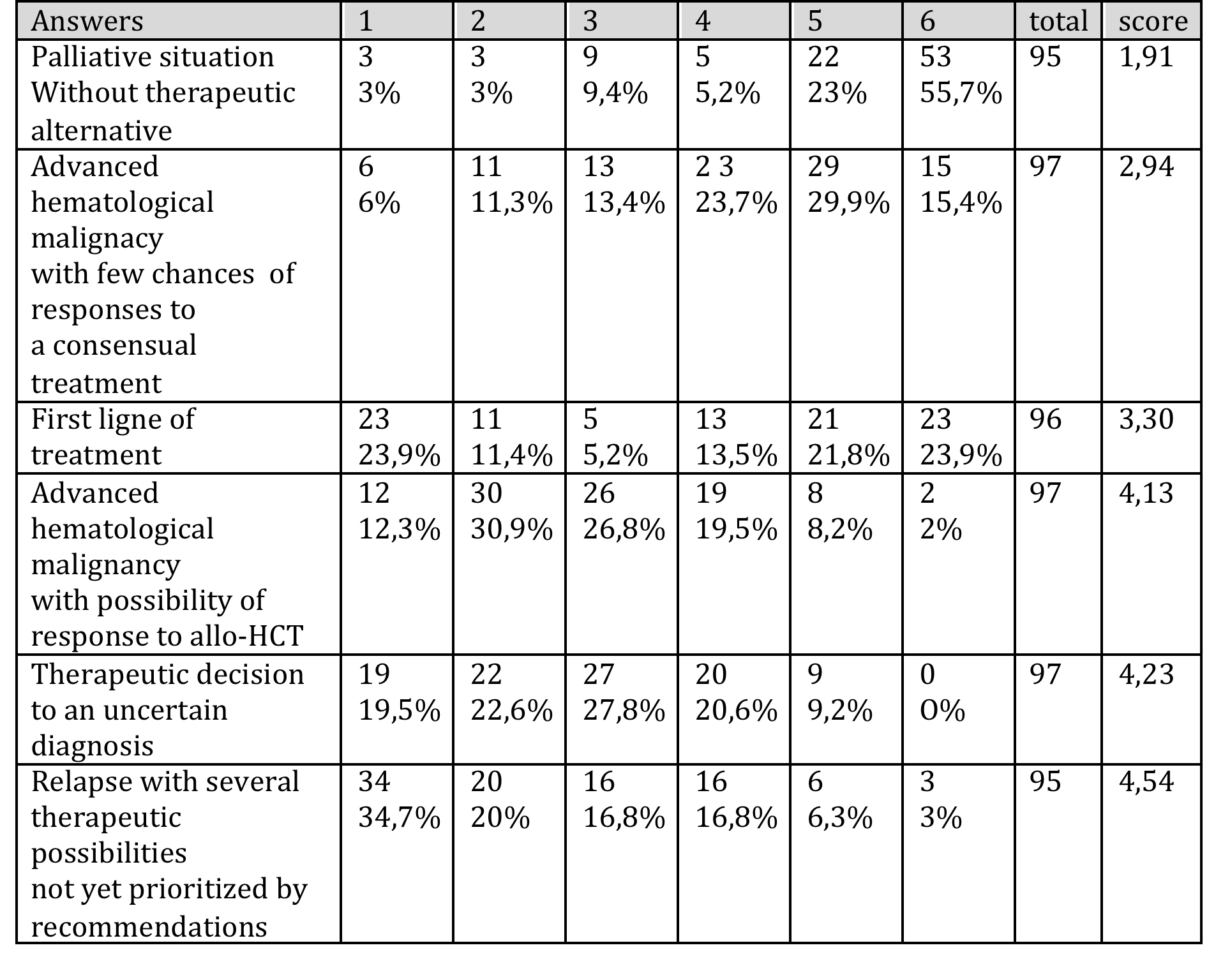 | | |
|  | | |
| **Information needed to arrive at the most appropriate therapeutic proposal** | | |
| Respondents were asked to rank proposed answers; averages were assigned to each answer choice (1= never, 5 = always) | | |
| 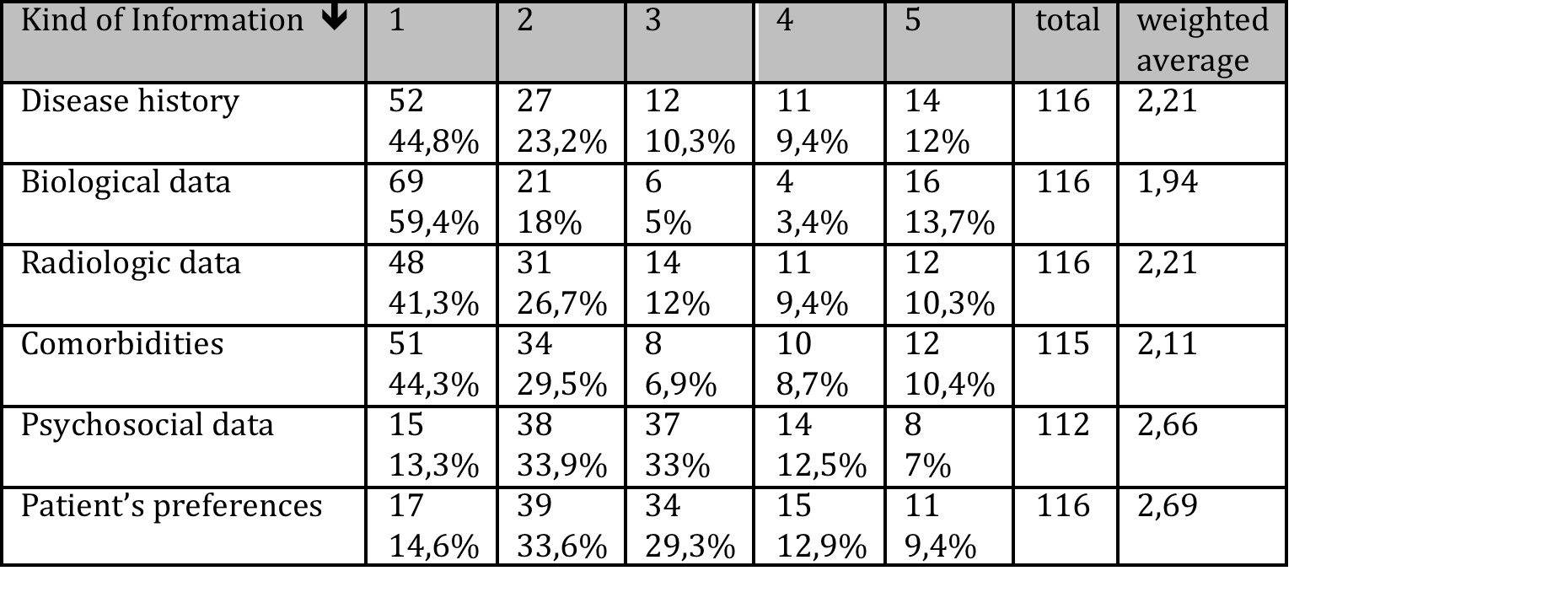 | | |
|  | | |
| **Reasons why MDTM may not be able to produce a therapeutic proposal** | | |
| Proposed Answers | 120 responses/ n. (%) | |
| Need to consult references or seek expert advice | 86 (72%) | |
| Lack of information regarding the pathology | 84 (70%) | |
| Lack of information regarding the patient | 68 (57%) | |
| Complexity of the case | 56 (47%) | |
| Disagreement among MDTM participants | 39 (32%) | |
| Lack of a clear question posed to the MDTM | 16 (13%) | |
| It never happens | 4 (3%) | |
|  | | |
| **The final word in case of divergent positions in MDTM** | | |
| Proposed Answers | 120 responses/ n. (%) | |
| Referent hematologist | 60 (51%) | |
| Head of the Department | 18 (15%) | |
| MDTM coordinator | 15 (13%) | |
| Patient | 1 (0,8%) | |
| other | 23 (19%) | |
|  | | |
| **Reasons for non-application of the MDtM therapeutic proposals** | | |
| Respondents were asked to rank proposed answers; averages were assigned to each answer choice (1= never, 5 = always) | | |
| 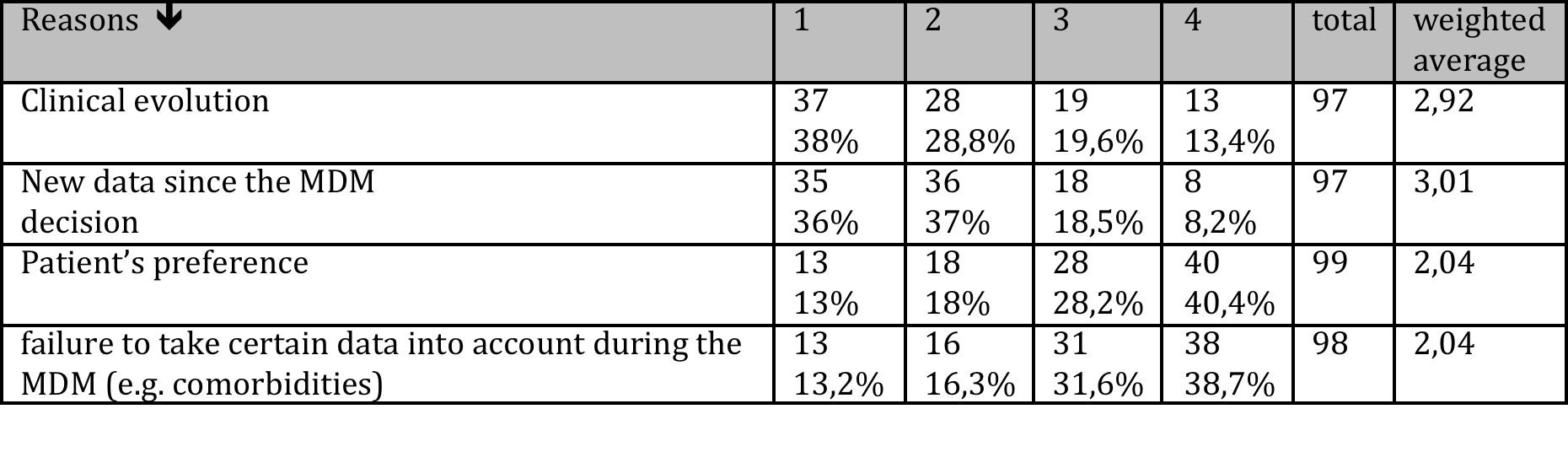 | | |
|  | | |
|  | | |
|  | | |
| **Attitudes if the treatment implemented is different from that proposed by the MDTM** | | |
| Respondents were asked to rank proposed answers; averages were assigned to each answer choice (1= never, 5 = always) | | |
| 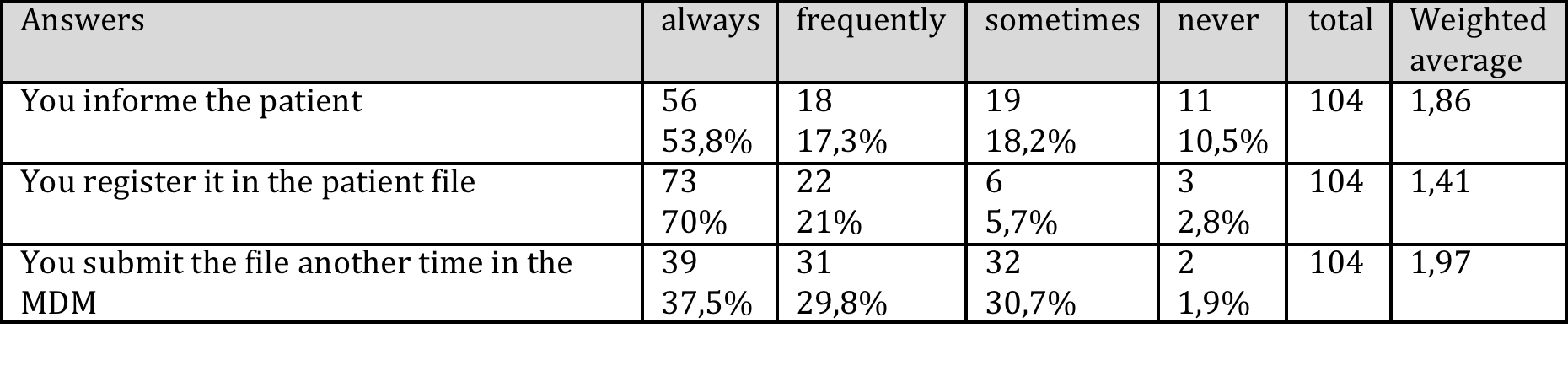 | | |
|  | | |
| **Respondent perceptions about benefits of MDTM for patients** | | |
|  | N (valid %-adjusting for missing data) | |
| Benefits of MDTM for patients | Strongly agree/agree | Disagree/ strongly disagree |
| Improving decision-making | 105 (100%) | 0 (0%) |
| Enhance decisions in line with the relevant guidelines | 100 (95%) | 5 (5%) |
| Improving quality of care | 94 (92%) | 9 (8%) |
| Feeling of safety for patients | 98 (92%) | 7 (8%) |
| Increase inclusion in clinical trials | 93 (91%) | 9 (9%) |
| Improving care coordination | 94 (89%) | 11 (11%) |
| Improving patients’ prognosis | 75 (73%) | 28 (27%) |
| Improve timeliness of exams or treatments | 42 (42%) | 60 (58%) |
| Enhance patient participation in medical decision-making | 37 (36%) | 67 (64%) |
|  | | |
| **Respondent perceptions about benefits of MDTM for professionals** | | |
|  | N, (valid %-adjusting for missing data) | |
| Benefits of MDTM for professionals | Strongly agree /agree | Disagree / strongly disagree |
| Information/knowledge sharing | 107 (100%) | 0 (0%) |
| Interactions with colleagues | 107 (100%) | 0 (0%) |
| Reviewing the patient file | 106 (99%) | 1 (1%) |
| Help with decision-making | 102 (98%) | 2 (2%) |
| Help in dealing with difficult situations | 96 (93%) | 7 (7%) |
| Reduced decision uncertainty | 97 (93%) | 7 (7%) |
| Shared legal responsibility | 94 (91%) | 9 (9%) |
| Help with communicating decisions to the patient | 88 (86%) | 15 (14%) |
| Improve work satisfaction | 87 (83%) | 18 (17%) |
| Time savings | 76 (73%) | 28 (27%) |
|  | | |
| **Respondent perceptions about disadvantages of MDTM** | | |
|  | N, (valid %-adjusting for missing data) | |
| Disadvantages of MDTM | Strongly agree /agree | Disagree / strongly disagree |
|  |  |  |
| Technical issues | 56 (55%) | 46 (45%) |
| Interrupts/delays | 53 (51%) | 51 (49%) |
| Exacerbation of problems within teams | 37 (36%) | 65 (64 %) |
| Inflationary proposal  (exams, consultations, treatments) | 35 (35%) | 66 (65%) |
| Time losses | 28 (27%) | 76 (73%) |
| Insufficient consideration of patient preferences | 26 (25%) | 76 (75%) |
| Peer judgments | 15 (15%) | 87 (85%) |
| Hierarchical decision-making without real exchange | 13 (12%) | 91 (88%) |
| MDTM is inappropriate in hematology | 7 (6%) | 95 (94%) |
| Our decisions can be challenged | 5 (5%) | 98 (95%) |
|  |  |  |
